# Combination of olfactory test and substantia nigra transcranial sonopraphy in the differential diagnosis of Parkinson’s disease: a pilot study from China

**DOI:** 10.1186/2047-9158-1-25

**Published:** 2012-12-26

**Authors:** Wei Chen, Yu-Yan Tan, Yun-Yun Hu, Wei-Wei Zhan, Li Wu, Yue Lou, Xi Wang, Yi Zhou, Pei Huang, Yuan Gao, Qin Xiao, Sheng-Di Chen

**Affiliations:** 1 Department of Neurology & Institute of Neurology, Ruijin Hospital affiliated to Shanghai Jiao Tong University School of Medicine, Shanghai, 200025, China; 2 Department of Ultrasound, Ruijin Hospital affiliated to Shanghai Jiao Tong University School of Medicine, Shanghai, 200025, China

**Keywords:** Parkinson’s disease, Hyposmia, Transcranial sonography

## Abstract

**Objectives:**

Both hyposmia and substania nigra (SN) hyperechogenicity on trascranial sonography (TCS) were risk markers for idiopathic Parkinson’s disease (PD), which was beneficial to the differential diagnosis of the disease. However, each of their single diagnostic value is often limited. The purpose of present study was to explore whether the combination of olfactory test and TCS of SN could enhance the differential diagnostic power in Chinese patients with PD.

**Methods:**

Thirty-seven patients with PD and twenty-six patients with essential tremor (ET) were evaluated on 16-item odor identification test from extended version of sniffin’ sticks and TCS of SN. The frequency of hyposmia and SN hyperechogenicity in each group was compared. The sensitivity, specificity, positive predictive value (PPV) and negative predictive value (NPV) of the two clinical biomarkers were analyzed.

**Results:**

The frequency of hyposmia in patients with PD was significantly higher than in patients with ET (62.2% *VS.* 3.8%, *P* = 0.000). The frequency of SN hyperechogenicity in patients with PD was significantly higher than in ET subjects (48.6% *VS.* 15.4%, *P* = 0.006). The combination of hyposmia and SN hyperechogenicity (if either one or both present) discriminated patients with PD from ET with a sensitivity of 78.4% and 29.7%, specificity of 80.8% and 100%, PPV of 85.3% and 100%, and NPV of 72.4% and 50.0%, respectively.

**Conclusions:**

Our preliminary data suggested that the combination of hyposmia and SN hyperechogenicity could improve the diagnostic potential for discriminating Chinese patients with PD from ET.

## Introduction

Parkinson’s disease (PD), especially in the early stage, is sometimes difficult to differentiate from essential tremor (ET) [1]. Accumulating evidence suggested that olfactory dysfunction, substantia nigra (SN) hyperechogenicity on transcranial sonography (TCS) might be risk markers of PD [2,3], which was helpful for making early and differential diagnosis. However, the diagnostic value of each item is often limited [4,5]. Recently, A few studies suggest that combination of TCS and hyposmia might play complementary roles for the discrimination of PD from ET and other movement disorders [6,7]. Since the prevalence of hyposmia in Chinese PD patients is lower than those in Western countries [8], whether the combination of hyposmia and SN hyperechogenicity could improve differential diagnostic value of Chinese patients with PD merits investigation.

Therefore, we conducted a pilot study to explore the value of combined olfactory test and TCS of SN for the differential diagnosis of PD in Shanghai, China.

## Patients and methods

### Subjects

Totally, thirty-seven patients with PD (mean age 63.8 ± 8.7 years, 11 women and 26 men), who fulfilled the UK PD brain bank criteria [9], were recruited from the movement disorders clinic at the Department of Neurology, Ruijin Hospital affiliated to Shanghai Jiao Tong University School of Medicine, Shanghai, China. None of the patients had undergone functional neurosurgery for PD. Twenty-six patients with ET were enrolled as disease controls (mean age 59.1 ± 12.3 years, 12 women and 14 men). Diagnosis of ET was established by consensus criteria [10]. For both groups, we excluded subjects with dementia determined by the Chinese version of the Mini-Mental State Examination (MMSE) [11], insufficient acoustic temporal bone window on TCS and those with possible olfactory influencing factors including nose surgery, nasal polyo, chronic sinusitis and acute upper respiratory tract infection. Clinical data, such as age, onset age, duration, disease stage (Hoehn and Yahr stage), motor severity (UPDRS III) and medications were collected from subjects. Written consents were also obtained. The study was approved by the Research Ethics Committee, Ruijin Hospital affiliated to Shanghai Jiao Tong University School of Medicine, Shanghai, China.

### Olfactory test

All participants underwent the 16-item odor identification test from extended version of sniffin’ sticks (SS-16; Burghart Messtechnik, Wedel, Germany). The examiner was blind to the diagnosis. As reported, the 16 odors in the original “Sniffin’ Stick” odor identification test were kept the same but alternative descriptions were slightly modified to accommodate for the Chinese population [8]. The descriptions “pine tree” and “grapefruit” were replaced with the Mandarin equivalent of “wood” and “pomelo”, which are common in mainland China and indicate the same or similar odors. In the present study, a SS-16 score < 9.5 was defined as hyposmia. This criterion was based on our prior study using the receiver operating characteristic curve (ROC) [8] . Both sensitivity and specificity were maximally high at this cut off value.

### Transcranial sonography

For TCS examination, a color-coded phase array ultrasound system(MyLab90, ESAOTE, Italy)with a 2.5 MHz phased-array transducer was used. The examination was done through the temporal bone window of the intact skull, scanning supratentorial and infratentorial brain areas in axial planes by tilting the probe. Special attention was paid to the mesencephalic brainstem. In the mesencephalic plane, the normal brainstem was visualized as a butterfly-shaped structure of low echogenicity. A structure was classified as hyperechogenic if the intensity of the ultrasound signal was abnormally increased, compared with a reference structure usually the surrounding white matter. An area of echogenicity ≤ 0.19 cm^2^ was classified as normal and areas of echogenicity ≥ 0.20 cm^2^ was classified as hyperechogenic. The examiners were ultrasound specialists blind to the clinical diagnosis.

### Statistics

Statistical analysis was performed with SPSS. Data were expressed as means ± SD. Independent-samples *t* test and chi-square analysis were employed for comparing continuous and categorical variables, respectively. Pearson correlation analysis was used for bivariate correlation. The significance level was set at *P* < 0.05.

## Results

Clinical characteristics of patients with PD and ET were shown in Table 1. There was no significant difference in age (*t* =1.663, *P* = 0.104) and gender (Pearson chi-square =1.777, *P* = 0.183) between the groups. Within the PD patients, 28 (75.7%) were in mild stage (H&Y 1-2), and 9 (24.3%) were in moderate to severe stage (H&Y 2.5-4). Mean disease duration was 3.6 years (SD = 3.1 years) and mean age of onset was 60.2 years (SD = 9.1 years). With respect to medications within the PD patients, 22(59.5%) were treated with levodopa, 7(18.9%) with dopamine agonist, 9(24.3%) with MAO-B inhibitor, 9(24.3%) with amantadine, 6(16.2%) with artane, and only 1(2.7%) with COMT inhibitor.

**Table 1:** Clinical characteristics of patients with PD and ET

	**PD (*n*** **= 37)**	**ET (*n*** **= 26)**	***P*value**
**Age, years**	63.8 ± 8.7	59.1 ± 12.3	0.104
**Gender (male/female)**	26/11	14/12	0.183
**Duration, years**	3.6 ± 3.1	12.0 ± 10.8	0.000
**MMSE**	27.5 ± 2.2	27.9 ± 1.4	0.407
**SS-16**	8.6 ± 2.9	12.2 ± 1.6	0.000
**UPDRS-III**	17.8 ± 12.3	-	
**H &Y stage**			
**1–2**	28	-	
**2.5–4**	9	-	
**Olfactory dysfunction**			0.000
**SS-16 < 9.5**	23	1	
**SS-16 > 9.5**	14	25	
**SN hyperechogenicity**			0.006
**Positive**	18	4	
**Negative**	19	22	

*PD* Parkinson’s disease, *ET* Essential tremor, *SS-16* the 16-item odor identification test from Sniffin’ Sticks, *SN* Substantia nigra.

### Hyposmia

The mean SS-16 score in patients with PD was 8.6 ± 2.9 (mean ± SD), which was significantly lower than that of ET (12.2 ± 1.6) (*t* = -5.630, *P* = 0.000). Based on the SS-16 score, hyposmia was observed in 23 patients with PD (62.2%) and 1 patient with ET (3.8%). The frequency of hyposmia in patients with PD was significantly higher than that in ET subjects (Pearson chi-square =22.020, *P* = 0.000). Among patients with PD, there was no significant correlation between the SS-16 score and disease duration (*r* = -0.121, *P* = 0.477) or motor severity (UPDRS III) (*r* = 0.117, *P* = 0.489).

### Substantia nigra hyperechogenicity

Eighteen patients with PD (48.6%) showed SN hyperechogenicity (7 unilaterally, 11 bilaterally), whereas four patients with ET (15.4%) exhibited SN hyperechogenicity (1 unilaterally, 3 bilaterally). The frequency of SN hyperechogenicity in patients with PD was significantly higher than that in ET subjects (Pearson chi-square = 7.435, *P* = 0.006). There was no significant difference in disease duration (*t* = -1.301, *P* = 0.202) or disease severity (UPDRS III) (*t* = -0.069, *P* = 0.945) between PD patients with SN hyperechogenicity and those without.

### Combination of the two biomarker features

There was no correlation between hyposmia and SN hyperechogenicity among patients with PD (Pearson chi-square = 0.302, *P* = 0.582). The sensitivity, specificity, positive predictive value (PPV), and negative predictive value (NPV) of the two tests (olfactory test, TCS of SN) were shown in Table 2. The combination of hyposmia and SN hyperechogenicity (if either one or both present) discriminated patients with PD from ET with a sensitivity of 78.4% and 29.7%, specificity of 80.8% and 100%, PPV of 85.3% and 100%, and NPV of 72.4% and 50.0%, respectively.

**Table 2:** Diagnostic features discriminating the patients with PD from ET

**Diagnostic feature**	**Sensitivity(%)**	**Specificity(%)**	**PPV(%)**	**NPV(%)**
(1) Hyposmia	62.2	96.2	95.8	64.1
(2) SN hyperechogenicity	48.6	84.6	81.8	53.7
(3) Either (1) or (2) present	78.4	80.8	85.3	72.4
(4) Both (1) and (2) present	29.7	100	100	50.0

*PD* Parkinson’s disease, *ET* Essential tremor, *PPV* Positive predictive value, *NPV* Negative predictive value, *SN* Substantia nigra.

## Discussion

This pilot study is the first to explore the value of combined olfactory test and TCS in the differential diagnosis of PD in China. Our preliminary data indicated that the combination of hyposmia and SN hyperechogenicity could improve the diagnostic potential for discriminating patients with PD from ET. If either hyposmia or SN hyperechogenicity was present, the sensitivity reached 78.4%, surpassing the corresponding value of each biomarker. Moreover, if both were found abnormal, the specificity was 100%, suggesting that this combination might be a feasible method for more accurate diagnosis of PD.

Our results suggested that hyposmia and SN hyperechogenicity might act as complementary roles for the discriminating of PD from ET. This phenomenon could be explained by several aspects. Firstly, from a pathophysiological perspective, they may be caused by different mechanisms. hyposmia in PD was associated with alpha-synuclein accumulation in central olfactory system, especially the olfactory bulb [12,13]. Whereas, SN hyperechogenicity may reflect increased SN iron content in PD, this was demonstrated in animal models and postmortem human brains [14,15]. Secondly, a few cross-sectional studies [6], as well as the present one, indicated that there was no obvious correlation between hyposmia and SN hyperechogenicity in PD. Thirdly, longitudinal follow-up studies suggested these two clinical biomarkers might reflect different prognosis. Baba and colleagues in Japan found that severe hyposmia in PD was a prominent clinical feature predicting the subsequent development of Parkinson’s disease dementia (PDD) [16]. While SN hyperechogenicity in PD was a stable finding, its area didn’t change during the course of disease [17]. Since these two items are independent risk factors for PD, both olfactory test and TCS could add supplementary information for the differential diagnosis.

To our knowledge, only a few studies have tried to explore the efficiency of combined two biomarkers for the early and differential diagnosis of PD. An observational study in Japan with small sample size found that the diagnostic sensitivity and specificity of olfactory test was 84.8% and 78.1%, and the corresponding value was 78.8% and 93.8% for TCS. If either one was present, the diagnostic sensitivity increased to 100% for discriminating patients with PD from normal controls [6]; Walter and co-workers in Germany evaluated the value of combined midbrain sonography, olfactory test and motor function assessment in the differential diagnosis of PD with larger sample size. They reported that, if both hyposmia and SN hyperechogencity were present, the diagnostic specificity and PPV reached to 89% and 95%, respectively [7]. Based on these studies, as well as the present one, it might be expected that combining olfactory test and TCS of the SN could provide a valuable method for the differential diagnosis of PD.

The present study has limitation because of its small sample size and the diagnosis of PD was clinical, without the evidence of functional neuroimaging [18]. The frequency of hyposima and SN hyperechogencity of PD in our study was lower than the patients in Western counties and Japan [3,5], which partly explains the low sensitivity of the two combined tests. Furthermore, the frequency of olfactory dysfunction in our ET patients was lower than that in German ET subjects [7]. These discrepancies might be attributable, at least to some extent, to recruited sample size and race diversity [19]. Selected bias might exist, since we enrolled subjects suitable for both olfactory tests and sufficient temporal bone window on TCS. This pilot study enrolled a relative low proportion of female patients with PD. In Asian subjects, the frequency of insufficient bone window on TCS was found to be higher than that in European counties, especially in elderly women [5,19]. Therefore, the true nature of the prevalence of SN hyperechogencity in Chinese patients with PD and this combination in the differential diagnosis of Chinese patients merit further investigation with larger sample size in future. As these two tests are easy to perform, noninvasive, and low-cost, the combination of these two tests is beneficial to further identification of the at-risk individuals who will develop PD, such as non-symptomatic first-degree relatives of PD and idiopathic REM sleep behavior disorders [20].

## Abbreviations

PD: Parkinson’s disease; SN: Substantia nigra; TCS: Transcranial sonography; SS-16: The 16-item odor identification test from Sniffin’ Sticks; PPV: Positive predictive value; NPV: Negative predictive value.

## Competing interests

The authors declare that they have no competing interests.

## Authors’ contributions

WC made contributions to conception and design, acquisition of data (statistical analysis) and in drafting the manuscript. Y-YT made contributions to conception and design, acquisition of data and in drafting the manuscript. LW, YL, XW, YZ, PH and YG participated in the execution and acquisition of data. Y-YH and W-WZ performed the TCS for the patients. S-DC and QX were the general supervisors of the research group, acquisition of funding, and involved in revising it critically for important intellectual content. All authors read and approved the final manuscript.
